# Conformational Changes in the Lower Palm Domain of ASIC1a Contribute to Desensitization and RFamide Modulation

**DOI:** 10.1371/journal.pone.0071733

**Published:** 2013-08-14

**Authors:** Erin N. Frey, Ryan E. Pavlovicz, Clem John Wegman, Chenglong Li, Candice C. Askwith

**Affiliations:** 1 Department of Neuroscience, Wexner Medical Center, The Ohio State University, Columbus, Ohio, United States of America; 2 Biomedical Sciences Graduate Program, The Ohio State University, Columbus, Ohio, United States of America; 3 Biophysics Graduate Program, The Ohio State University, Columbus, Ohio, United States of America; 4 Division of Medicinal Chemistry, College of Pharmacy, The Ohio State University, Columbus, Ohio, United States of America; University of Houston, United States of America

## Abstract

Acid-sensing ion channel 1a (ASIC1a) is a proton-gated cation channel that contributes to fear and pain as well as neuronal damage following persistent cerebral acidosis. Neuropeptides can affect acid-induced neuronal injury by altering ASIC1a inactivation and/or steady-state desensitization. Yet, exactly how ASIC1a inactivation and desensitization occur or are modulated by peptides is not completely understood. We found that regions of the extracellular palm domain and the β_11-12_ linker are important for inactivation and steady-state desensitization of ASIC1a. The single amino acid substitutions L280C and L415C dramatically enhanced the rate of inactivation and altered the pH-dependence of steady-state desensitization. Further, the use of methanethiosulfonate (MTS) reagents suggests that the lower palm region (L280C) undergoes a conformational change when ASIC1a transitions from closed to desensitized. We determined that L280C also displays an altered response to the RFamide peptide, FRRFamide. Further, the presence of FRRFamide limited MTS modification of L280C. Together, these results indicate a potential role of the lower palm domain in peptide modulation and suggest RFamide-related peptides promote conformational changes within this region. These data provide empirical support for the idea that L280, and likely this region of the central vestibule, is intimately involved in channel inactivation and desensitization.

## Introduction

ASICs are proton-gated, voltage-insensitive cation channels expressed throughout the nervous system [1–4]. ASICs contribute to pain, sensory transduction, fear-related behaviors, anxiety, and depression [3,5–11]. ASIC1a also causes neuronal death following prolonged extracellular acidosis within the central nervous system [12,13]. To date, acidosis and ASIC1a-mediated neuronal dysfunction have been implicated in stroke, multiple sclerosis, and amyotropic lateral sclerosis [12,14–16]. Acidosis-mediated neuronal death is limited by induction of steady-state desensitization in ASIC1a [17]. Steady-state desensitization occurs during slow or incremental changes in pH that are not sufficient to provoke robust activation of the channel [18]. Desensitized ASICs fail to activate, even when significantly more acidic solutions are encountered. Compounds that enhance ASIC1a desensitization inhibit ASIC1a-mediated neuronal death and compounds that prevent desensitization can exacerbate death [13,17]. Most ASICs also inactivate and conduct current only for a short time after activation. Steady-state desensitization and inactivation are often intertwined [19–21]. Inactivation has been implicated in both sustained acid-dependent responses (pain) and ASIC’s role in behavior [22,23].

Peptides can alter ASIC gating and impact ASIC-dependent behaviors and neuronal damage [7,10,11,13,17,21,24–33]. The first identified peptide modulators of ASICs were RFamide-related peptides (RFamides), which end in arginine-phenylalanine-amide [24]. RFamides inhibit both inactivation and steady-state desensitization of ASICs [21,24–28,34–36]. Thus, RFamides prolong the length of time that ASICs conduct current in response to an acidic stimulus and allow ASIC1a activation under more acidic basal conditions [21,24,26,28,37].

**Figure 1:**
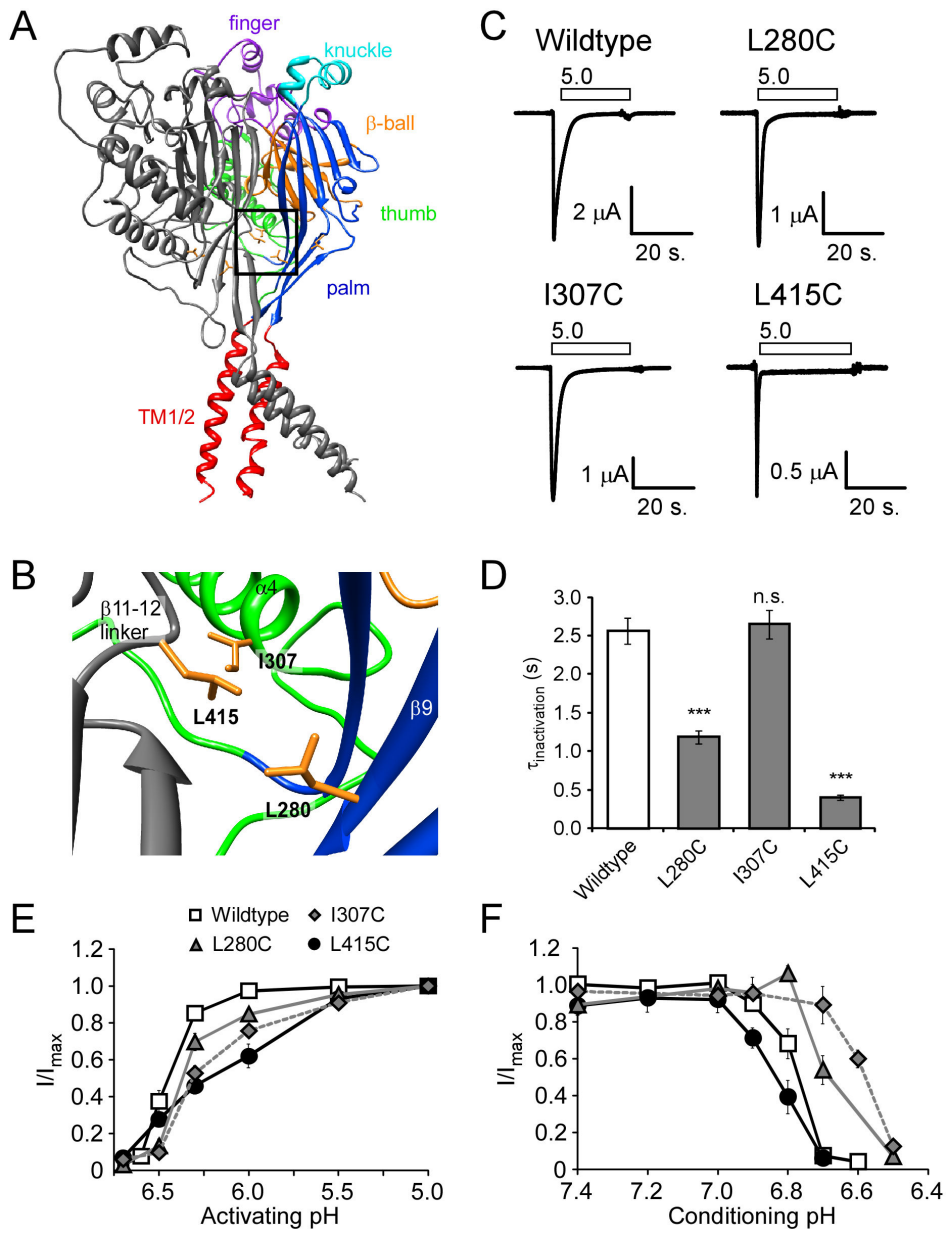
Location and characteristics of L280C, I307C, and L415C in human ASIC1a. **A**–**B**. Human ASIC1a was modeled based on the chicken ASIC1 crystal structure (PDB ID: 3HGC). One subunit has been removed to show the inside of the central vestibule. The subunits are color-coded to highlight different regions of the ASIC1a structure. The boxed region is magnified in **B** to illustrate the positions of L280, I307, and L415. Images were rendered using the UCSF Chimera package [63,64]. **C**. Representative recordings of acid-activated currents in *Xenopus* oocytes expressing wild-type human ASIC1a, L280C, I307C, or L415C. Basal pH was maintained at pH 7.4 before application of pH 5.0 (white bars above trace). **D**. Quantification of the tau of inactivation (*n* = 10-14), calculated through a single exponential fit of the decay phase of the acid-evoked current. **E**. Quantification of proton-dependent activation (*n* = 6-9). I/I_max_ is the peak current amplitude evoked from test pH conditions normalized to peak current amplitude evoked with pH 5.0. **F**. Quantification of the proton-dependence of steady-state desensitization (*n* = 6-10). I/I_max_. is the peak current amplitude of pH 5.0-evoked currents after conditioning in the test pH normalized to the pH 5.0-evoked current after conditioning in pH 7.9 (see methods). Data are mean ± SEM. “***” indicates a *p*-value < 0.001, respectively. “n.s.” indicates no significant difference.

Most peptide modulators of ASICs are thought to interact with the large extracellular domain to modify the gating characteristics of the channels. The crystal structure of chicken ASIC1 shows a trimeric channel with a large funnel shaped extracellular domain [38–41]. The extracellular domain of each desensitized subunit resembles a closed hand with a wrist, palm, thumb, finger, and knuckle as well as a “β-ball” domain situated between the thumb, finger, and knuckle domains [39] (illustrated in Fig. **1A**). In this manuscript, we examine the role of L280 within the β-strand 9 (β9) of the lower palm domain (Fig. 1A, B) in steady-state desensitization, inactivation, and RFamide modulation. Residues within the extracellular loop immediately adjacent to this region and residues within the upper palm domain on the alternate side of β9 have been previously implicated in RFamide modulation [28]. Multiple studies have also suggested that the palm domain plays a critical role in ASIC inactivation and steady-state desensitization [28,41–43]. Conformational changes within the β-linker regions associate with the desensitized state. Specifically, L415 (in the linker region) interacts with L280 (lower palm) and I307 (thumb) when the channel is desensitized (Fig. 1B) [41]. Using site-directed mutagenesis and the substituted cysteine accessibility method, we examined the role of L415, L280, and I307 on channel inactivation and steady-state desensitization. Our data suggests that L280 and L415 play an intimate role in channel desensitization/inactivation. Further, RFamide modulation of ASIC1a gating likely involves conformation changes within this area.

## Methods

### Modeling Human ASIC1a

A homology model of the human acid-sensing ion channel 1a (hASIC1a) was created using the X-ray crystal structure of the chicken ASIC1a (PDB ID: 3HGC, 3.0 Å, pH 6.5) [38]. Based on the template structure, we were able to model residues 45-452 which includes the transmembrane and extracellular domains. Two hundred models were built with the automodel function of MODELLER 9v8 [44,45]. Human ASIC1a contains a two residue insertion in the β9-α4 loop of the thumb domain (residues 298-299) that is not present in the chicken structure. This insertion was incorporated into the modeling process in which the final model was selected based on the MODELLER DOPE score [46]. Although the sequence identity is high between the template and target structures (~90% identity and ~95% similarity in the modeled regions), due to the medium resolution of the modeling template, PROCHECK was used to further assess the quality of the model [47]. The PROCHECK results indicated that the model is of ‘good’ quality based on 91.4% of the 1086 non-glycine, non-proline amino acids falling into the most favored regions of the Ramachandran plot [47]. When including the additionally allowed regions of the Ramachandran plot, the percentage of well-modeled residues increases to 99.4%. Only one amino acid was found in a disallowed region. This model was used to illustrate residues and regions of interest of human ASIC1a (Figure 1A, B). For clarity, all ASIC numbering and nomenclature refers to human ASIC1a.

### Expression of Recombinant DNA in Xenopus Oocytes

The Stratagene QuikChange® site-directed mutagenesis kit (Cedar Creek, TX) was used to introduce point mutations into the human ASIC1a cDNA (GENBANK accession number NM_001095) within the pMT3 expression vector. Mutations were confirmed by DNA sequencing at the Plant-Microbe Genomics Facility at The Ohio State University and high quality plasmid DNA was prepared for injection using Qiagen (Valencia, CA) Midi prep kits. Female *Xenopus laevis* were purchased from *Xenopus I* (Dexter, MI) and *Xenopus* oocytes were isolated using standard procedures [28]. This work was implemented in accordance with the Guide for the Care and Use of Laboratory Animals of the National Institutes of Health and approved by the Institutional Animal Care and Use Committee of The Ohio State University (protocol # 2009A0161-R1). The oocyte removal surgery was performed under tricaine methanesulfonate (MS222) anesthesia and all efforts were made to minimize suffering. Ovarian lobes were removed and digested for 1.5-2 hours at room temperature with 1.2 mg/ml collagenase in Modified Barth’s solution lacking calcium (24 mM NaHCO_3_, 88 mM NaCl, 15 mM HEPES, 1 mM KCl, 0.8 mM MgSO_4_, 125 units/liter penicillin/streptomycin) to separate individual oocytes and remove follicular membranes. Healthy stage IV and V oocytes were selected and maintained in Modified Barth’s solution containing calcium (0.4 mM CaCl_2_ and 0. 3 mM Ca(NO_3_)_2_). The nuclei of isolated oocytes were injected with ~5 ng of a 100 ng/µl stock of wildtype or mutant ASIC1a expression plasmids using a PV820 pneumatic picopump (World Precision Instruments, Sarasota, FL). Oocytes were incubated at 18^° C^ for 18-96 hours before experiments were performed.

### Ethics Statement

This work was implemented in accordance with the Guide for the Care and Use of Laboratory Animals of the National Institutes of Health and approved by the Institutional Animal Care and Use Committee of The Ohio State University (protocol # 2009A0161-R1). *Xenopus laevis* oocyte removal surgery was performed under tricaine methanesulfonate (MS222) anesthesia and all efforts were made to minimize suffering.

### Electrophysiology

The two-electrode voltage clamp technique was used to measure whole cell currents from *Xenopus* oocytes heterologously expressing ASIC1a channels as described [28,48]. Electrodes were pulled using a Sutter P-97 micropipette puller (Sutter Instrument Co., Novato, CT) and filled with 3 M KCl (pipette resistance of 0.5–2 MΩ). Oocyte recordings were done in a modified RC-Z3 250 µl oocyte recording chamber (Warner Instruments, Hamden, CT). Data were acquired using an Oocyte Clamp OC-725 Amplifier (Warner Instruments, Hamden, CT) and a Powerlab 4SP digitizer with CHART5 software (ADI Instruments, Colorado Springs, CO). Except where noted, the extracellular solution contained (in mM): 116 NaCl, 2 KCl, 5 HEPES, 5 MES, 2 CaCl_2_, 1 MgCl_2_, with pH adjusted using 1N NaOH. The solution exchange rate in the recording chamber was ~1 ml/s. The holding potential was -60 mV unless otherwise noted. [2-(Trimethylammonium)ethyl] Methanethiosulfonate Bromide (MTSET) was purchased from Toronto Research Chemicals, Inc. (Ontario, Canada). FRRFamide peptide (FRRFa) and FRRF (no amide) were synthesized from EZBiolab, Inc. (Westfield, IN).

In order to obtain the pH dependence of activation, basal pH was maintained at pH 7.4. Maximal current was established by rapid application of saturating acidic solution (pH 5.0) to the oocyte. Peak current amplitude was measured for each test pH and normalized to the average peak amplitude of flanking pH 5.0 currents to minimize the impact of potential tachyphylaxis. For measurements of the pH dependence of steady-state desensitization, oocytes were maintained in a basal solution of pH 7.9. Test conditioning pHs were applied to the oocytes for 2 minutes prior to activation with pH 5.0. After activation cells were washed with pH 7.9 and allowed to recover for at least 30 seconds: a time that was experimentally verified to allow maximal recovery for all channels used in this study. Maximal current was defined as the peak current amplitude evoked by pH 5.0 after the cells were conditioned with pH 7.9. As before, maximal currents were measured before and after test conditioning pH to minimize the effect of possible tachyphylaxis of proton-gated current. Wildtype ASIC1 currents are generally transient and inactivate completely. However, under some conditions a sustained acid-dependent current persists after the transient current. This residual current was quantified by measuring the current remaining 60 seconds after transient activation (I_residual_) and (where applicable) normalized to the transient peak current amplitude (I_transient_).

### MTSET Experiments

Oocytes were maintained at a basal pH of 7.4 and maximal currents were established with application of pH 5.0. Currents were allowed to decay for at least 60 s. The control residual current was determined by normalizing the current remaining at 60 s to the peak amplitude (I_residual_/I_transient_). After recovery, 300 µM MTSET was applied at pH 7.4 for the indicated duration and excess MTSET was washed away with at least 10 mL of pH 7.4 solution. The pH-dependence of activation and steady-state desensitization were measured as described above. During experiments using MTSET, the effect on residual current decreased slightly over time. Specifically, the effect of MTSET modification of L280C displayed modest run down with repeated pH 5.0 applications with peak amplitude (5% decrease, *n* = 8, *p* = 0.02 compared to control, paired Students t-test), residual current (17 ± 5% decrease, *n* = 8, *p* = 0.003 compared to control, paired Students t-test), and the inactivation rate (31 ± 9% decrease, *n* = 8, *p* = 0.01, paired Students t-test) decreasing. In order to control for this phenomenon, all oocytes received the same number of washes over the same amount of time before residual current data were collected. Treatment with MTSET did not alter inactivation characteristics of wildtype ASIC1a (τ_inact_ = 1.8 ± 0.2 s before MTS and 1.8 ± 0.2 s after MTS, *n* = 3, *p* = 0.9; % residual current = 0.16 ± 0.05% before MTS and 0.31 ± 0.04% after MTS, *n* = 3, *p* = 0.3; and [49]). Furthermore, MTSET did not alter activation with pH 6.5 or steady-state desensitization with pH 6.7 of wildtype ASIC1a (activation pH_6.5/5.0_ = 66 ± 10% before MTS and 62 ± 3% after MTS, *n* = 3, *p* = 0.7; conditioning pH_6.7/7.4_ = 1.8 ± 0.7% before MTS and 1.5 ± 0.6% after MTS, *n* = 3, *p* = 0.2).

### Kinetics of MTSET Modification

In order to assess the accessibility of L280C and L415C, MTSET was applied while the channel was either in the closed or desensitized state. Since MTSET modification itself is pH-dependent, we utilized the Ca^2+^-dependence of ASIC1a to identify a single pH where individual channels could be closed or desensitized-depending on the Ca^2+^ concentration of the solutions [42,50,51]. For closed-state analysis, oocytes were maintained in modified Ringers solution containing 5 mM CaCl_2_. For desensitized-state kinetics, oocytes were maintained in modified Ringers solutions containing no added calcium. During the experiments, oocytes were incubated at pH 6.8 or 6.9 for 2 minutes as indicated. Then 300 µM MTSET was applied for the indicated time at pH 6.8 or 6.9. After MTSET modification, excess MTSET was washed away with 10 mL pH 6.8 or 6.9. Then oocytes were washed with 10 mL pH 7.4. Residual current was assessed as described above.

**Figure 3:**
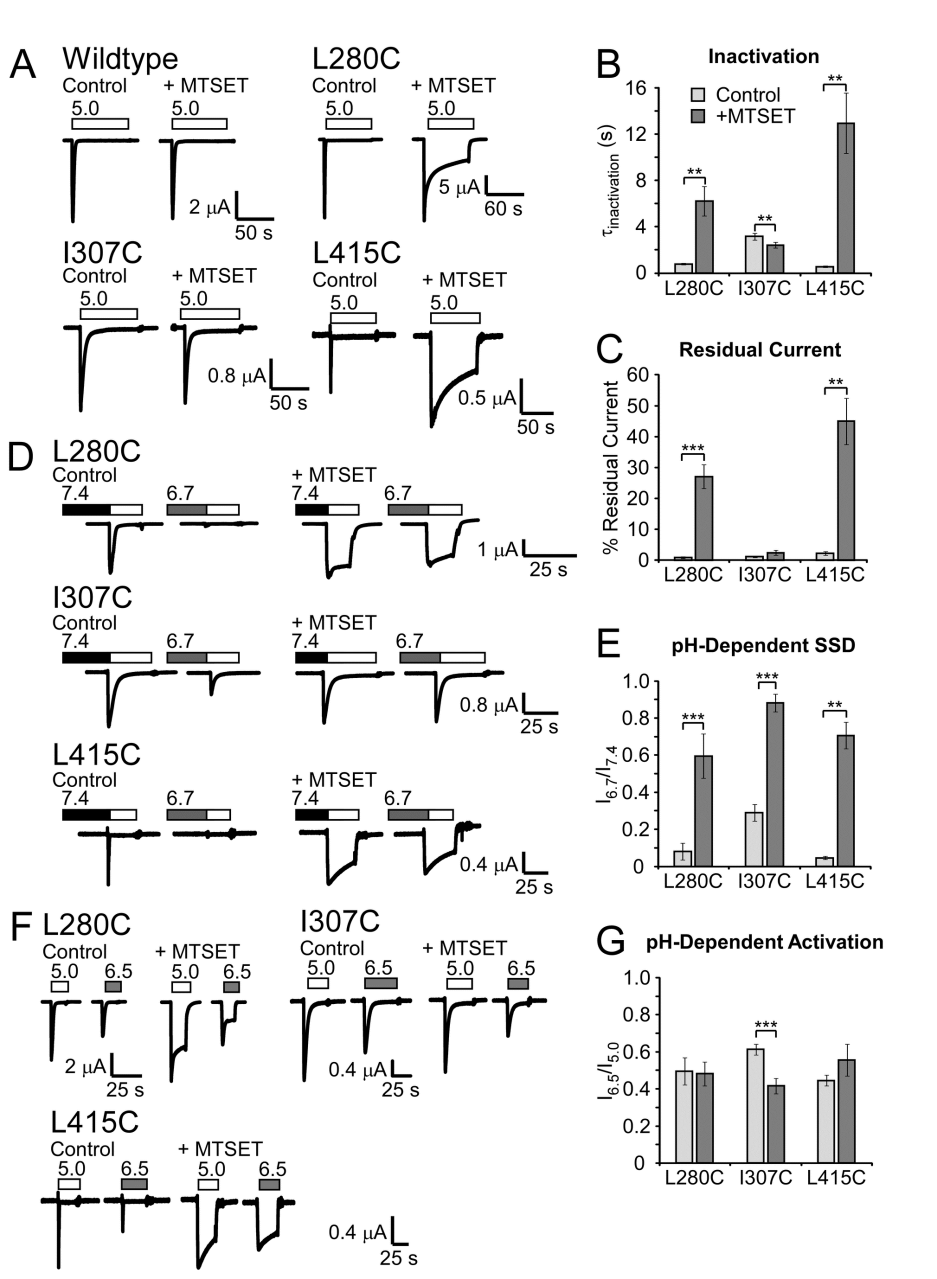
MTSET modification of desensitized L280C and L415C. **A**. Representative traces of calcium-dependent gating of unmodified L280C and L415C. Oocytes were exposed to the indicated pH (pH 6.8 for L280C or pH 6.9 for L415C) in either 5.0 mM Ca^2+^ or nominal Ca^2+^ -free solutions to foster ASIC1a transition to distinct gating states at the same pH. **B**. Quantification of calcium-dependent gating. The presence of 5.0 mM Ca^2+^ maintained L280C and L415C in the closed state when cells were treated with the conditioning pH (pH 6.8 for L280C or pH 6.9 for L415C) for ~2 minutes. This was evident by robust pH 5.0 evoked currents following conditioning with the mildly acidic pH (*n* = 6-8). Conditioning with the same pH in nominal Ca^2+^ induced steady-state desensitization of L280C and L415C (*n* = 6-8). **C**. Schematic of the experiment to assess accessibility. The closed state of ASIC1a was maintained by conditioning with pH 6.8 (L280) or 6.9 (L415) containing 5 mM Ca^2+^. The desensitized state was evoked by conditioning with the same pH in nominal calcium. 300 µM MTSET was applied in the conditioning pH for the indicated time and then excess MTSET was washed away with conditioning solution. Oocytes were then returned to pH 7.4 before activation with pH 5.0. **D**. Representative traces of L280C pH 5.0-evoked currents before (control) and after 6 minutes of MTSET incubation**. E**. Quantification of residual current of L280C after MTSET exposure for the indicated time (*n* = 4-6). **F**. Representative traces of L415C before (control) and after 60 second intervals of MTSET incubation. **G**. Quantification of residual current of L415C after MTSET exposure for the indicated time (*n* = 4-6). Curves represent one-phase association exponential fits of the data points. Data are mean ± SEM. “***” indicates *p* < 0.001 compared to 5.0 mM Ca^2+^ conditioning.

### Peptide Modulation

100 µM FRRFa or FRRF (no amide) was applied to oocytes at a holding pH 7.4 for 1 minute before activation with pH 5.0. Residual current was measured 1 minute following activation and was normalized to peak current amplitude, as described above. In order to assess peptide effect on steady-state desensitization, 100 µM FRRFa was applied to oocytes at holding pH 7.4 for 1 minute followed by pH 6.7 for 2 minutes. Then, proton-gated currents were evoked with pH 5.0. The peak current amplitude evoked after conditioning was normalized to flanking pH 5.0-evoked currents conditioned with pH 7.4 (also containing FRRFa).

### Peptide-MTSET Competition

Vehicle or 100 µM peptide was applied for 1 minute at pH 7.4. Then pH 7.4 solution containing 300 µM MTSET and vehicle or peptide was applied for the indicated time. After MTSET modification, excess MTSET was washed away with pH 7.4 solution containing vehicle or peptide. Before residual current was measured, oocytes were washed with at least 50 mL of pH 7.4 over 10 minutes in order to remove peptides. H_2_O was the vehicle for FRRFamide and DMSO was the vehicle for FRRF (no amide). No difference was observed between H_2_O and DMSO vehicle, so data were collectively pooled as “vehicle.”

### Data Analysis

Data were analyzed using CHART5 software (ADInstruments) and Igor Pro (WaveMetrics, Inc. Lake Oswego, OR). To measure the rate of channel inactivation, the decay phase of the current was fitted to a single exponential equation:

$$I=k_{0}+k_{1}e^{-t/\tau inact}$$

and the τ_inact_ was calculated. In order to calculate the τ_inact_ following peptide modulation or MTSET modification, the region from the peak to ~30 seconds after activation was fit with a single exponential. In some oocytes, inactivation kinetics displayed a bi-exponential time course following modulation with FRRFa or MTSET. However, this effect was highly variable. The pH_0.5_ for activation and desensitization were calculated by fitting the data from individual oocytes using the equation:

$$I/I_{pH}{}_{\left(\max\right)}=1/(1+{\left(EC_{50}/[H^{+}]\right)}^{n})=1/(1+10^{n}{}^{\left(pH-pH0.5\right)})$$

where *n* is the Hill coefficient, and EC_50_ and pH_0.5_ are the proton concentration and pH that elicit half of the saturating peak current amplitude. In order to calculate the FRRFa EC_50_, this equation was adjusted so that EC_50_ represented the half maximal dose of FRRFa and pH_0.5_ was replaced with the log of the FRRFa concentration needed to evoke a half maximal response. 100 µM FRRFa induced maximal response in both wildtype and L280C channels. Thus, FRRFa responses at each concentration were normalized to the response with 100 µM FRRFa. Unpaired, two-tailed Student’s t tests were used unless otherwise indicated. A “*p*” value less than 0.05 was considered significant.

## Results

### L280C, I307C, and L415C alter ASIC1a gating

Expression of L280C (Leucine 280 to cysteine), I307C, and L415C in *Xenopus* oocytes produced appreciable acid-gated channels (Figure 1; Table 1). Yet, L280C and L415C displayed faster inactivation kinetics (as indicated by a smaller τ_inactivation_) compared to wild type channels (Figure 1C, D; Table 1). L415C also displayed a small, yet significant, increase in the residual current remaining after the transient phase (Figure 1C; Table 1). These results suggested that L280 and L415 play a role in channel inactivation. However, I307C did not show such differences in inactivation characteristics (Figure 1C, D; Table 1). The pH required for half-maximal activation (pH _0.5_Act) was smaller in all three mutant channels by at least 0.11 pH unit (Figure 1E; Table 1). These results indicate that more acidic pH values were required to activate the channel. However, these mutations had divergent effects on apparent proton sensitivity of steady-state desensitization (SSD). While L280C and I307C showed half maximal desensitization at more acidic pHs (~0.1-0.2 pH units), L415C desensitized at slightly more basic pH values (Figure 1F; Table 1). Thus, mutations L280C, I307C, and L415C maintain the basic mechanisms of ASIC activity, although the characteristics of the channels are slightly perturbed.

**Table 1:** Characteristics of cysteine Mutants.

	**pH_0.5_Act**	**pH_0.5_SSD**	**τ_inactivation_(s)**	**Residual Current (µA)**	**Peak Current Amplitude (µA)**
**Wildtype**	6.47 ± 0.02 (8)	6.79 ± 0.01 (9)	2.56 ± 0.2 (14)	-0.032 ± 0.008 (15)	-6.6 ± 0.9 (15)
**L280C**	6.36 ± 0.02 (6)**	6.67 ± 0.03 (6)***	1.18± 0.09 (10)***	-0.043 ± 0.005 (10)	-4.1 ± 0.7 (10)
**I307C**	6.23 ± 0.04 (6)***	6.60 ± 0.03 (7)***	2.65 ± 0.2 (26)	-0.051 ± 0.009 (13)	-4.1 ± 0.5 (13)*
**L415C**	6.20 ± 0.06 (6)***	6.84 ± 0.01 (7)*	0.40 ± 0.03 (11)***	-0.059 ± 0.008 (10)*	-2.4 ± 0.3 (11)***

Characteristics of L280C, I307C, and L415C compared to human ASIC1a. Table shows the pH_0.5_Act (pH of half maximal activation), pH_0.5_SSD (pH of half maximal steady-state desensitization-SSD), the tau of inactivation, amplitude of residual currents, and peak current amplitude. Data represent the mean +/- SEM. The “n” value is indicated in parenthesis. Statistical significance was determined using the Student’s t-test compared to wildtype ASIC1a. "*”, “**”, and “***” indicates a *p* < 0.05, 0.01, and 0.001, respectively.

### MTSET modification of L280C, I307C, and L415C, alters inactivation and steady-state desensitization

**Figure 2:**
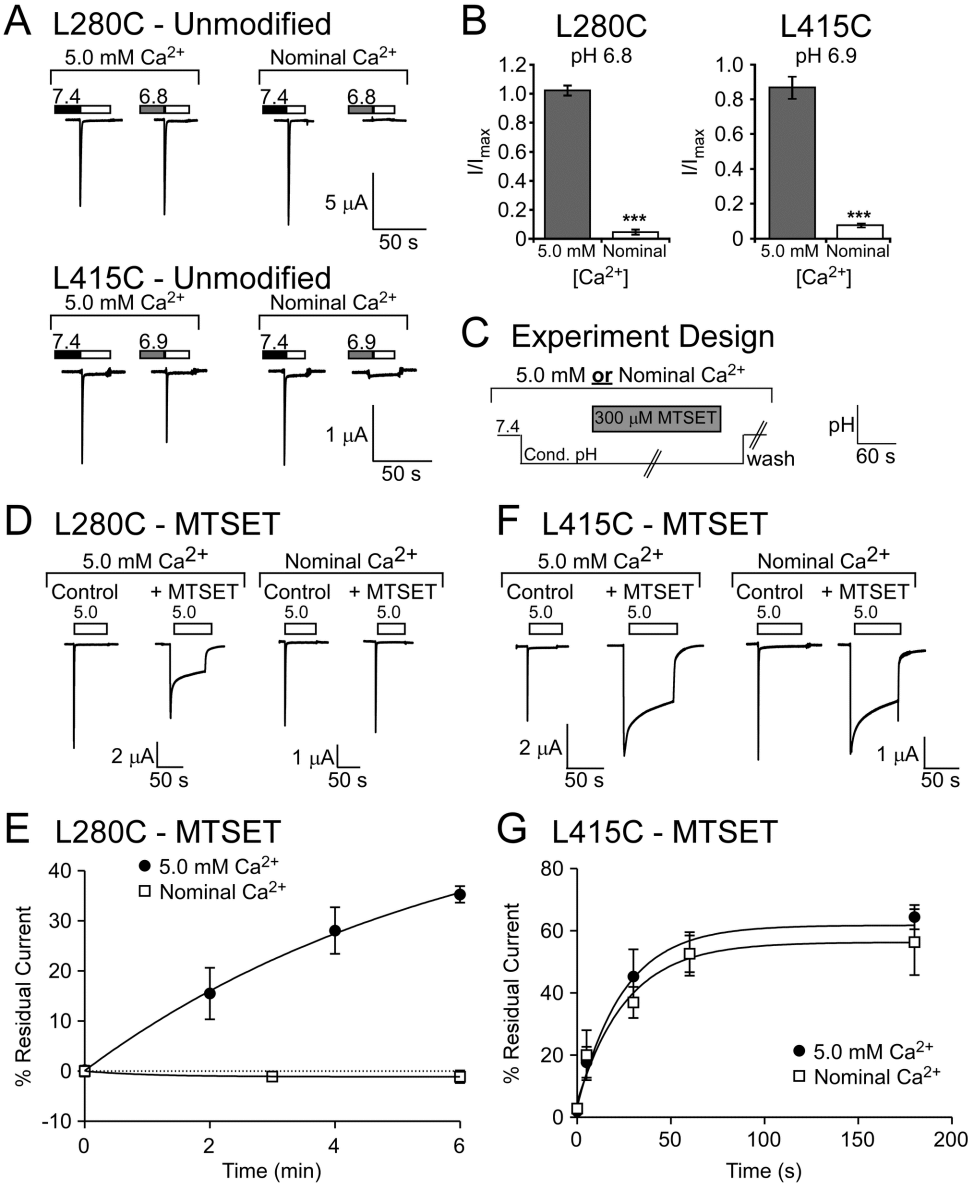
MTSET modification of L280C, I307C, and L415C. **A**. Effect of MTSET on wildtype or mutant ASIC1a expressed in *Xenopus* oocytes. MTSET (300 µM) was applied at pH 7.4 for 3 minutes and removed by washing with pH 7.4 solutions. pH 5.0-evoked currents after MTSET incubation (“+ MTSET”) were compared to control currents in the same oocyte measured before MTSET application (“Control”). **B**–**C**. Quantification of (**B**) tau of inactivation (*n* = 6-8) and (**C**) residual current (*n* = 6-7). **D**. Representative recordings of steady-state desensitization (SSD) before and after MTSET modification. Oocytes were maintained at a basal of pH 7.9 and then incubated with pH 6.7 for 2 minutes to induce SSD (shaded bars) prior to activation with pH 5.0 (white bars). **E**. Quantification of MTSET-dependent changes in SSD (*n* = 5-6). **F**. Representative traces of MTSET exposure on pH-dependent activation. MTSET was applied as above and the response to pH 5.0 (white bars) or pH 6.5 (gray bars) from basal pH 7.4 was measured. **G**. Quantification of pH 6.5-mediated activation before and after MTSET modification (*n* = 6-8). Data are mean ± SEM. “**” and “***” indicate *p*-values < 0.01 and 0.001, respectively. Significance was determined with paired Student’s t-tests.

Covalent modification of free cysteine residues by MTS-related chemicals is a useful tool to probe the importance of specific amino acid positions in ion channel gating. To further examine the roles of L280C, I307C, and L415C in channel gating, the cationic MTS reagent, MTSET was applied at pH 7.4. After treatment, excess MTSET was washed away and channel characteristics were examined. Similar to previous reports, MTSET exposure did not alter wildtype ASIC1a inactivation, activation with pH 6.5, or steady-state desensitization with pH 6.7 (Figure **2** and Methods) [49]. However, MTSET modification of L280C and L415C slowed the rate of inactivation and increased residual current (Figure 2A–C). These results strongly suggest that MTSET modification at position 280 and 415 inhibits ASIC1a inactivation. Similar effects were not observed with MTSET modification of I307C, which showed a small increase in the rate of inactivation after MTSET exposure (Figure 2B). MTSET modification of all three mutants resulted in robust current after conditioning with pH 6.7, indicating SSD was inhibited by MTSET modification as well (Figure 2D, E). MTSET modification decreased the relative response of I307C to activation with pH 6.5 suggesting the pH dependence of activation was also affected in this mutant (Figure 2F, G). Together, these results confirm a role for L280, I307, and L415 in ASIC1a gating. In particular, modifications of L280C and L415C impaired both inactivation and steady-state desensitization. This result, combined with the effects of mutagenesis of these residues, strongly supports the idea that L280 and L415 play a particularly important role in desensitization and inactivation. Further investigations focused on the involvement of L280 and L415 in conformation changes associated with inactivation and desensitization.

### L280C modification is state dependent

The inactivated and desensitized states are thought to represent the same or very similar ASIC conformations [19]. Studies suggest there is a conformational change as the channel transitions between the closed state and the inactivated/desensitized state [42,52]. Thus, changes in accessibility of particular residues might be expected between the closed and desensitized/inactivated state [42]. Since MTSET robustly affects L280C and L415C, we determined the apparent accessibility of MTS during different gating states. The MTS reaction is inherently pH sensitive, being slower at more acidic pHs [50]. However, ASIC1a gating is affected by both pH and calcium [19,53]. Therefore, we identified a pH for L280C (pH 6.8) and L415C (pH 6.9) that would maintain the closed state or promote desensitization through alterations in the extracellular calcium concentration (Figure 3A, B) [42]. To assess MTSET accessibility, channels were exposed to the appropriate pH and calcium condition, MTSET was applied for the indicated time, and then MTSET was washed away in the conditioning solution before cells were returned to pH 7.4 (Figure 3C). Channels were then activated and MTSET-dependent changes in residual current were analyzed. In the 5.0 mM Ca^2+^ conditioning solution, which maintains the channel in the closed state, MTSET modification of L280C was observed within 2 minutes as indicated by the increase in residual current (Figure 3D, E). In the nominal Ca^2+^-free conditioning solution, which maintains the channel in the desensitized state, MTSET did not appreciably modify L280C even after 6 minutes (Figure 3D, E). This suggests that MTSET modification of L280C is dependent on the conformational state of the channel and accessibility of L280C is attenuated when the channel is desensitized. In contrast, accessibility of L415C did not appear to be state-dependent (Figure 3F). Indeed, MTSET modification occurred with similar rates regardless of whether the L415C channel was in the closed or desensitized state (Figure 3G). Thus, our data support the idea that the β-linker region containing L415C is readily accessible in multiple gating states. However, the lower palm domain region around L280 becomes inaccessible to MTSET when the channel is desensitized.

### L280C responds to FRRFa

**Figure 4:**
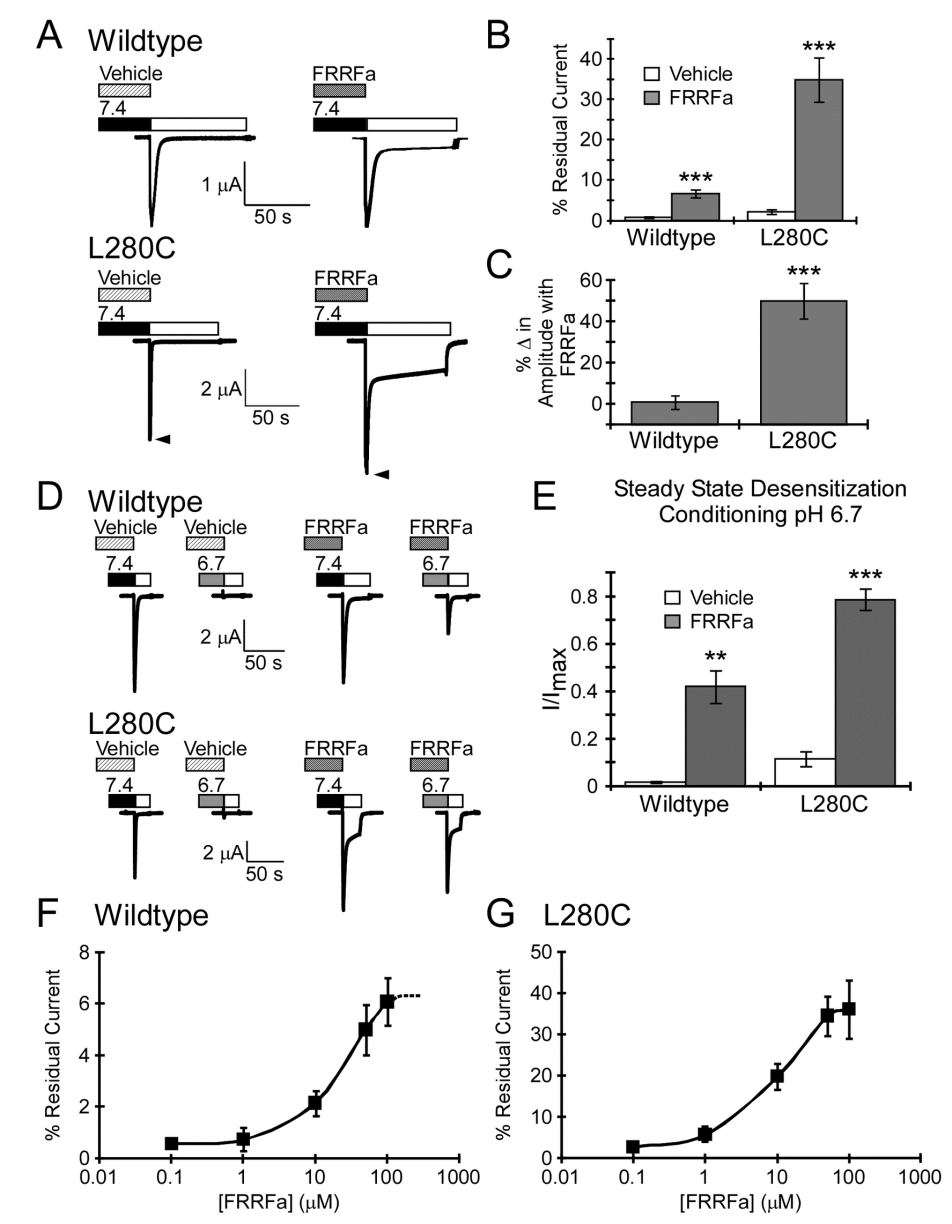
FRRFa modulation of L280C. **A**. FRRFa increases residual current of wildtype and L280C. FRRFa (100 µM) was applied for 1 minute at pH 7.4 prior to activation with pH 5.0. For L280C, arrowheads highlight the FRRFa-induced increase in peak current amplitude. **B**. Quantification of residual current (*n* = 14-17). **C**. Quantification of FRRFa modulation on pH 5.0-evoked peak current amplitude. The percent change in amplitude was determined by subtracting the pH 5.0-evoked peak current amplitude of vehicle from the pH 5.0 evoked peak current amplitude evoked after FRRFamide modulation and normalizing to the vehicle peak current amplitude from the same cell (*n* = 17-25). **D**. Representative trace of FRRFa modulation on steady-state desensitization. 100 µM FRRFa or vehicle was applied for 1 minute at basal pH 7.4 and again during the 2 minute incubation with conditioning pH 6.7. Proton-gated current was evoked with pH 5.0 (white bar). **E**. Quantification of FRRFa modulation of steady-state desensitization (*n* = 4). **F**–**G**. FRRFamide concentration response curve for wildtype ASIC1a (**F**) and L280C (**G**). The effect of FRRFamide on residual current was assessed. Our data suggest that 100 µM FRRFa induced a maximal response on wildtype ASIC1a as 300 µM FRRFa was not significantly different from 100 µM FRRFa (*n* = 5, *p* = 0.69 paired Student’s t-test, difference between 100µM and 300µM was 10.42% ± 11.25%; *data not shown*). Based on this information, the calculated EC_50_ for wildtype ASIC1a was 20 ± 4 µM and 14 ± 4 µM for L280C (*n* = 6-8, *p* = 0.34). Data are mean ± SEM. “**” and “***” indicates *p*-value < 0.01 and 0.001 respectively.

RFamide-related peptides limit both inactivation and steady-state desensitization of wildtype ASIC1a in a mechanism which may be dependent on the gating state of the channel [24,28]. Due to the gating dependence of MTSET modification of L280C and the fact that MTSET modification of L280C resembles RFamide modulation, we investigated the role of this residue in FRRFamide modulation of ASIC1a (Figure 4). These experiments used the synthetic peptide, FRRFamide (FRRFa), since this small peptide mimics endogenous RFamides and produces robust RFamide modulation. The relative change in inactivation with FRRFamide was larger in L280C compared to wildtype ASIC1a (wildtype ASIC1a: Control τ_inact_ = 2.3 ± 0.2 s; FRRFamide τ_inact_ = 3.2 ± 0.2 s; 45 ± 6% increase, *n* = 14: L280C: Control τ_inact_ = 0.94 ± 0.07 s; FRRFamide τ_inact_ = 2.0 ± 0.1s; 131 ± 16% increase, *n* = 17; *p* = 6x10^-5^ compared to wildtype ASIC1a with FRRFa). FRRFa also increased residual current and the peak current amplitude of L280C (Figure 4A–C). FRRFa impaired steady-state desensitization of both wildtype ASIC1a and L280C (Figure 4D, E). In addition, the calculated EC_50_ of FRRFa modulation was not statistically different between wildtype and L280C (Figure 4F, G). Together, these results suggest that the enhanced results with FRRFa on L280C residual current are due to peptide efficacy.

### FRRFa modulation occludes L280C from MTSET

**Figure 5:**
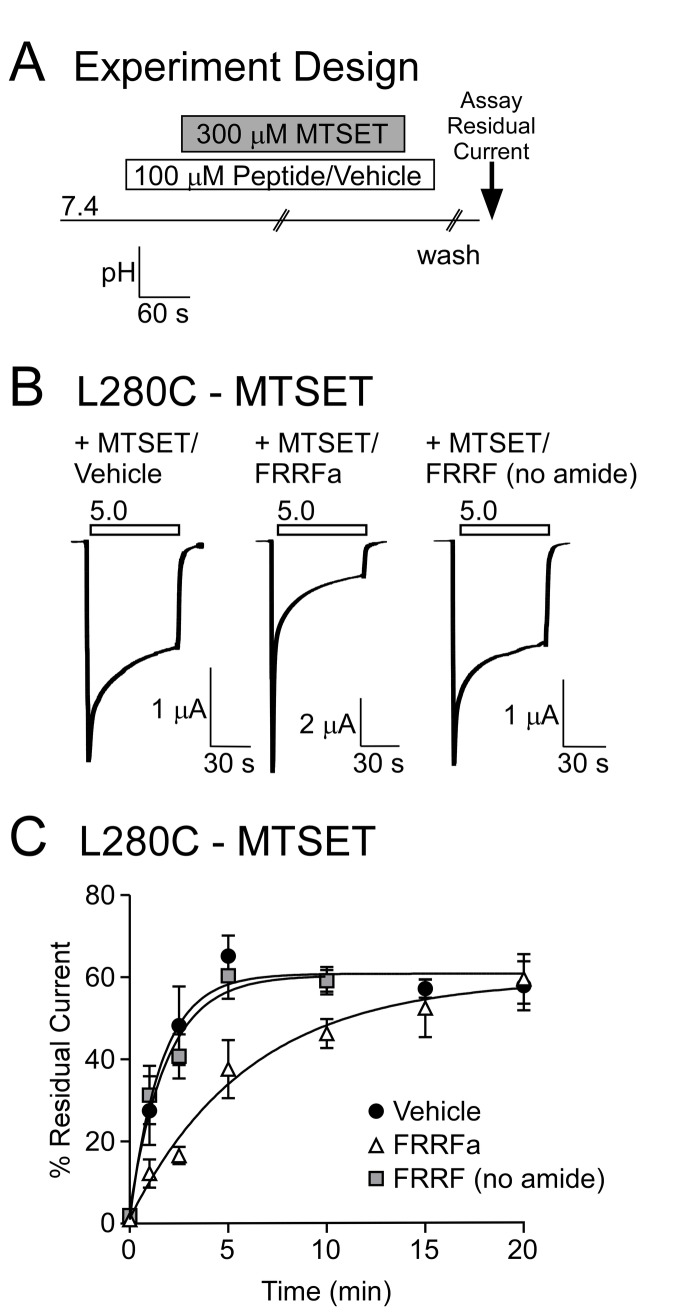
FRRFa impairs MTSET modification. **A**. Schematic of MTSET/peptide experimental design. 100 µM peptide or vehicle was applied for 1 minute before application of MTSET (in the presence of vehicle or peptide). Excess MTSET was removed by washing in peptide (or vehicle) containing solutions. Finally, peptide (or vehicle) was washed-out thoroughly and residual current was measured. **B**. Representative traces of L280C pH 5.0-evoked currents following 2.5 minutes of MTSET modification in the presence of vehicle, FRRFa, or FRRF (no amide). **C**. Quantification of residual current after exposure to MTSET in the presence of vehicle, 100 µM FRRFa, or 100 µM FRRF (no amide). Curves represent one-phase association exponential fits of the data points. FRRFa significantly altered MTSET modification (*p* < 0.001, 2-way ANOVA). No difference was observed between Vehicle and FRRF (no amide). Data are mean ± SEM, *n* = 3-22.

In order to further examine if L280 is involved in peptide modulation, we set out to determine if FRRFa could affect accessibility of L280C. Channels were pretreated with vehicle or FRRFa and then incubated with MTSET (still in the presence of vehicle or FRRFa) for the indicated time at pH 7.4 (Figure 5A). Multiple washes were then performed, first to remove excess MTSET (washed with pH 7.4 containing vehicle or FRRFa) and then to remove FRRFamide (pH 7.4 solution). Channels were activated with pH 5.0 and MTSET modification was assessed by measuring residual current. MTSET incubation in the presence of FRRFa resulted in a smaller MTSET-mediated change in residual current compared to vehicle (Figure 5B). In the presence of FRRFa, the maximal MTSET effect was not obtained for 15 minutes, whereas the MTSET modulation plateaued after 5 minutes in the presence of vehicle (Figure 5C). The one-phase association curves were also significantly different between vehicle and FRRFa (*p* < 0.001, 2-way ANOVA). These data suggest that FRRFa limits MTSET accessibility for L280C. The peptide FRRF lacking the c-terminal amide was used as a control, as the c-terminal amide of RFamide-related peptides is central to modulation of ASIC1a. Consistent with this, the residual current of L280C with 100 µM FRRF (no amide) was substantially smaller (5.6 ± 0.8%) than residual current with FRRFa (*p* = 2x10^-6^). FRRF (no amide) did not interfere with MTSET modification (Figure 5B, C). Thus, the limiting effect of FRRFa correlates with the ability to modulate ASIC1a.

### FRRFa modulates MTSET modified L280C

**Figure 6:**
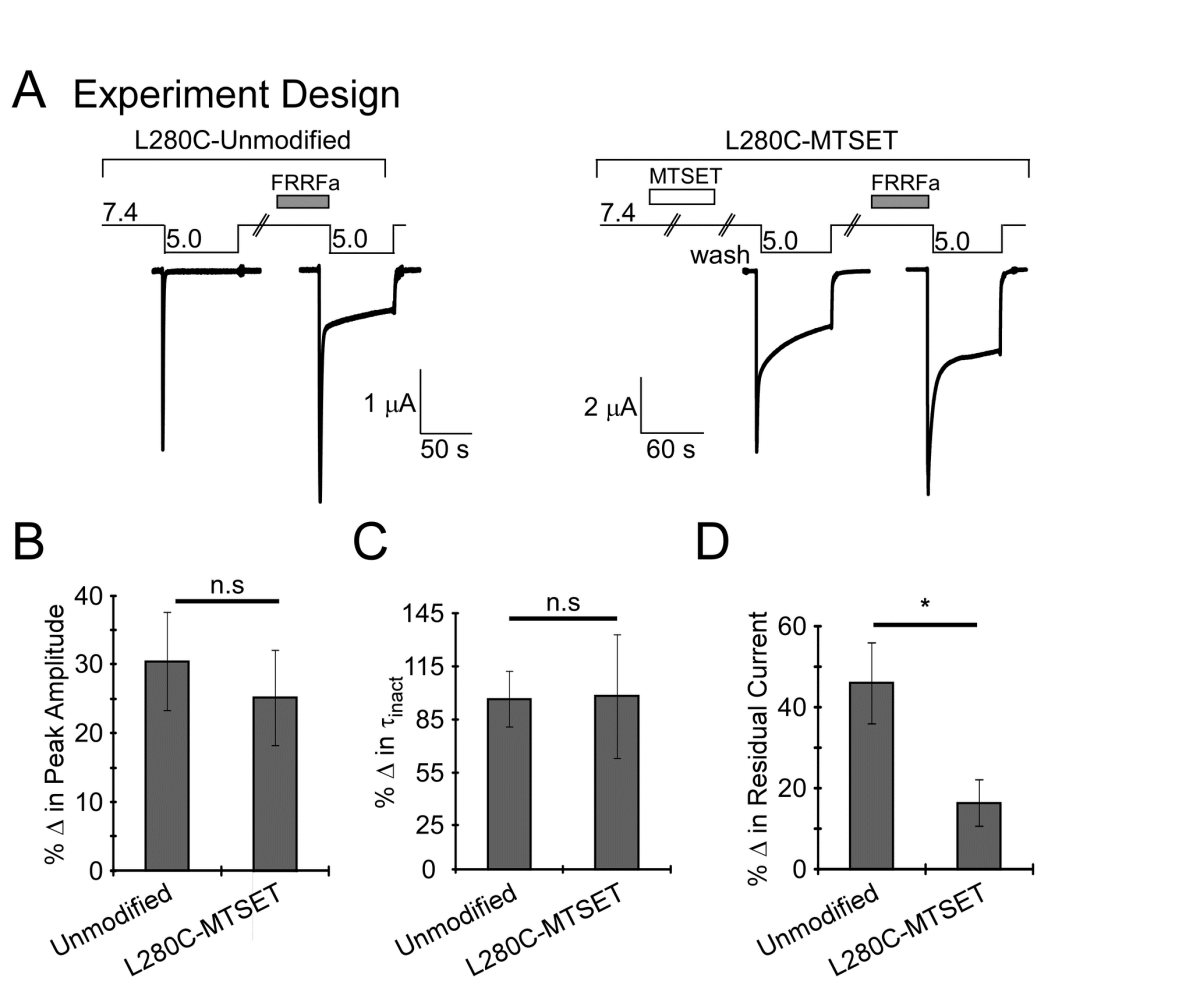
FRRFa modulates L280C after MTSET modification. **A**. Schematic of experimental design and representative traces. Left Panel: L280C was activated with pH 5.0 with and without 100 µM FRRFa. Right Panel: L280C was modified with 300 µM MTSET for 3 minutes at pH 7.4. After treatment, MTSET was removed by washing with pH 7.4 and pH 5.0-evoked current was recorded in the absence of FRRFamide. Channels were then washed with pH 7.4 and allowed to recover for 1 minute. Then pH 7.4 solutions containing 100 µM FRRFa were applied for 1 minute. After application of FRRFa, channels were activated with pH 5.0. For quantification (**B**–**D**), % change was determined by subtracting the stated characteristic (peak amplitude, residual current, or τ_inact_) with FRRFa from control (no peptide) and normalized to the no peptide response. **B**. Quantification of % change in peak current amplitude. The magnitude of the change in peak current amplitude evoked with FRRFa was independent of MTSET modification (*n* = 8-11, *p* = 0.6). **C**. Quantification of the % change in the rate of inactivation (τ_inact_). FRRFa response on inactivation after MTSET was not significantly different from FRRFa response on unmodified L280C (*n* = 8-11, *p* = 0.9). **D**. Quantification of % change in residual current. After MTSET modification, FRRFa still increased residual current (*n* = 8, *p* = 0.02, paired Student’s t-test), but this was not as robust as FRRFa-induced residual current of unmodified L280C (*n* = 8-11, *p* = 0.03). Data are mean ± SEM. “*” indicates *p* < 0.05 and n.s. indicates no significant difference.

FRRFa could reduce MTSET accessibility for L280C indirectly by promoting a conformational change. Alternatively, FRRFa could reduce the accessibility of MTSET through a mechanism which involves direct interaction with the amino acid position 280. Under such conditions, we might expect that L280C modified with MTSET would be insensitive to FRRFa (as MTSET mimics FRRFa modulation). However, there was no significant difference between FRRFa modulation of peak current amplitude or the τ_inact_ between modified and unmodified L280C (Figure 6A–C). The effect of FRRFa on the residual current of MTSET modified L280C was reduced compared to unmodified L280C (Figure 6D). However, the reduction in residual current might be due a “ceiling” effect given the already large residual current. Together, these results indicate that MTSET modulation of L280C does not prevent FRRFa modulation and suggests that FRRFa occludes MTSET access to L280C through an indirect mechanism.

## Discussion

Inactivation and desensitization are powerful mechanisms that limit ASIC1a activity. Inactivation controls the length of time ASIC1a conducts current after acidification. Steady-state desensitization (SSD) controls whether ASIC1a is receptive to activation at a specific basal pH [18]. Insights into inactivation and desensitization have been provided by electrophysiology experiments with mutant channels as well as crystallography of chicken ASIC1a. Many of these experiments suggest that the lower palm domain as well as the β1-β2 and β11-β12 linker regions play pivotal roles in gating [39,41–43,54–58]. Our data provide support for the role of the lower palm domain (L280) and the β11-β12 linker region (L415) in ASIC gating.

The β1-β2 and β11-β12 linker regions are proposed to undergo specific conformational changes with desensitization [41,56–58]. For clarity, all ASIC numbering and nomenclature within this discussion refers to the analogous position in human ASIC1a. Desensitization brings A81 in the β1- β2 linker and V414 in the β11-12 linker in close proximity and, when mutated to cysteines, formation of a disulfide bond between these residues locks the channel into the desensitized state [57]. Furthermore, the conformation of both linker regions is distinctly different in the desensitized cASIC1a crystal structure compared to the open-high pH PcTx1-bound structure [39,41]. The open structure (PDB ID: 4FZ0) shows L415 of the β11-β12 linker interacting with L85 of the β1-β2 linker [41]. In the desensitized structure, L415 makes hydrophobic contacts with I307 at the base of α4 of the thumb domain, V367 within β10 of the lower palm domain, and L280 within β9 of the lower palm domain [39,41]. We show that mutation of L415 and L280 to cysteines affects ASIC1a channel inactivation and steady-state desensitization. Similarly, modification of these mutants with MTSET dramatically slows inactivation and inhibits steady-state desensitization. These results support a role for L415 and L280 in desensitization gating. Mutation of L415C within the β11-β12 linker also decreases the apparent proton-sensitivity for activation but increases sensitivity for steady-state desensitization. Interestingly, mutation of L85, which is shown to interact with L415 in the open state of the channel, also has divergent effects on proton-dependent gating [41,56]. Thus, our data provide support that disruption of this interaction affects pH-dependent gating. We also find that the rate of MTS modification of L415C is not altered when the channel is closed versus desensitized. This result is somewhat surprising given the large conformational differences of the L415 side chain observed in the high pH and low pH crystal structures [41]. However, our results support the idea that conformational changes in the β11-β12 linker are critical for channel gating as disruption of both L415 and L280 disrupts desensitization and inactivation.

### Constriction of the Lower Palm Domain in Inactivation/Desensitization

L280 is located within the palm domain, a region composed of β-sheets that form the structural backbone of ASIC channels [39]. Together, these β-sheets line the central vestibule of the ASIC channel [38,39]. The side chain of L280 projects into the central vestibule (Figure 1B). Our data indicated that modification of L280C is state dependent, with MTSET modification occurring efficiently when the channel is closed, but not when the channel is desensitized. These results confirm that conformational changes in this region occur with gating and suggest that L280 (and perhaps the central vestibule) are more accessible in the closed state than the desensitized state. L280 is in close proximity to other residues within the palm domain involved in inactivation/desensitization. Specifically, modification of E79C (located in β1 within the lower palm domain) prevents inactivation in the same manner observed for L280C and L415C [42]. Like L280, the side chain of E79 also projects into the central vestibule. Further, modification of E79C with MTS reagents is state-dependent, with E79C being inaccessible when the channel is in the desensitized state [42,43]. E79 and E418 are thought to contribute directly to conformational transitions in the lower palm domain during inactivation by coordinating a proton at acidic pH [41]. This is thought to lead to constriction of the lower palm domain [41–43]. Our data with L280 support the idea that such conformational changes within the lower pore domain accompany channel gating.

### RFamide Modulation and the Lower Palm Domain

In neurons, RFamides slow inactivation, enhance residual or sustained current, increase peak current amplitude, and decrease the apparent pH sensitivity of steady-state desensitization [21,24–26,28,34,36]. Slowing inactivation and producing a sustained residual current would allow ASICs to generate a sustained response to acidic pH. Limiting steady-state desensitization would allow ASICs to respond during slower, more gradual pH declines that would normally desensitize the channel. Co-localization and the convergence of functional data suggest that the interaction between RFamides and ASICs could be particularly important for fear or pain [37]. Indeed, some of the effects of RFamide-related peptides have been hypothesized to involve ASIC-like channels [59]. However, the concentration of RFamides required for ASIC modulation is high and would likely be encountered only within microdomains such as the synaptic cleft [3,24]. More importantly, no direct evidence has been reported that endogenous RFamides modulate ASIC activity *in vivo*. Yet, RFamide modulation provides a unique tool toward understanding how ASICs desensitize and inactivate as well as how extracellular modulators can affect these properties.

RFamide modulation of ASIC channels is thought to occur through direct interaction between RFamides and the extracellular domain of the channel [21,24,26,28]. RFamides require significant time to mediate alterations in ASIC current suggesting modulation involves a slow RFamide-mediated conformational change or access of RFamides to their binding site is limited by more factors than simple diffusion. It is hypothesized that FMRFamide binds well to the closed state of the channel and dissociates very slowly from the desensitized state [24]. RFamides and calcium might compete for action of ASICs, although it is clear that RFamides do not act simply by removing calcium [21]. Yet, the specific region in which RFamides bind to the channel and how this impacts channel gating is unknown. Previously, we reported that residues within β9 and the loop connecting β9 to α4 of the thumb are critical for FMRFamide modulation of human ASIC1a [28]. Data suggested, however, that these regions were involved in transducing the signal of RFamide interaction to changes in channel gating and did not affect binding itself. Here, we find that the presence of FRRFa hinders MTSET modification of L280 suggesting FRRFa binding results in a conformational change that affects this region of the palm domain, possibly to slow desensitization/inactivation. This supports the finding that transitions in the palm domain are involved in transducing changes from the extracellular modulators to changes in channel gating. Interestingly, GMQ activates ASIC3 and produces non-desensitizing currents by interacting with residues in the lower palm domain of ASIC3 including E79 [60,61]. GMQ functions as a gating modifier to modulate ASIC3 activity and acts in part by preventing pH-dependent desensitization [62]. Thus gating modifiers that prevent contraction of the lower palm domain contribute to non-inactivating acid-induced currents [41,60,61]. Our results suggest that RFamide modulation involves conformational changes within the lower palm domain that hinder inactivation and desensitization to induce residual currents in ASIC1a. Together, our results highlight the interaction between the lower palm and the β11-β12 linker region in channel inactivation and desensitization and suggest that compounds can modulate these characteristics by affecting conformational changes within the lower palm.
